# Reducing ppGpp Level Rescues an Extreme Growth Defect Caused by Mutant EF-Tu

**DOI:** 10.1371/journal.pone.0090486

**Published:** 2014-02-28

**Authors:** Jessica M. Bergman, Disa L. Hammarlöf, Diarmaid Hughes

**Affiliations:** Department of Medical Biochemistry and Microbiology, Uppsala University, Uppsala, Sweden; University of British Columbia, Canada

## Abstract

Transcription and translation of mRNA's are coordinated processes in bacteria. We have previously shown that a mutant form of EF-Tu (Gln125Arg) in *Salmonella* Typhimurium with a reduced affinity for aa-tRNA, causes ribosome pausing, resulting in an increased rate of RNase E-mediated mRNA cleavage, causing extremely slow growth, even on rich medium. The slow growth phenotype is reversed by mutations that reduce RNase E activity. Here we asked whether the slow growth phenotype could be reversed by overexpression of a wild-type gene. We identified *spoT* (encoding ppGpp synthetase/hydrolase) as a gene that partially reversed the slow growth rate when overexpressed. We found that the slow-growing mutant had an abnormally high basal level of ppGpp that was reduced when *spoT* was overexpressed. Inactivating *relA* (encoding the ribosome-associated ppGpp synthetase) also reduced ppGpp levels and significantly increased growth rate. Because RelA responds specifically to deacylated tRNA in the ribosomal A-site this suggested that the *tuf* mutant had an increased level of deacylated tRNA relative to the wild-type. To test this hypothesis we measured the relative acylation levels of 4 families of tRNAs and found that proline isoacceptors were acylated at a lower level in the mutant strain relative to the wild-type. In addition, the level of the *proS* tRNA synthetase mRNA was significantly lower in the mutant strain. We suggest that an increased level of deacylated tRNA in the mutant strain stimulates RelA-mediated ppGpp production, causing changes in transcription pattern that are inappropriate for rich media conditions, and contributing to slow growth rate. Reducing ppGpp levels, by altering the activity of either SpoT or RelA, removes one cause of the slow growth and reveals the interconnectedness of intracellular regulatory mechanisms.

## Introduction

Translation Elongation Factor Tu (EF-Tu) plays a crucial role in protein synthesis [1], forming a complex with each aminoacylated tRNA and carrying it to the decoding site on translating ribosomes. The degree of saturation of elongating ribosomes by ternary complex (EF-Tu·GTP·aa-tRNA) is a major determinant of the maximum growth rate of bacteria [2]. In *Salmonella enterica* subsp. *enterica* serovar Typhimurium strain LT2 (hereafter referred to as *S.* Typhimurium) EF-Tu is encoded by two widely separated genes, *tufA* and *tufB*, that encode identical proteins [3], [4]. Each gene can be individually inactivated without lethal effect [5]. Strains in which one *tuf* gene is inactivated produce approximately 66% of the wild-type amount of EF-Tu and have a maximum growth rate in rich medium (Luria broth, LB) that is reduced to a similar degree [2], [3], [6]. Strains in which only one *tuf* gene is present (or active) facilitate the study of the phenotypes associated with mutant variants of EF-Tu.

We have previously shown that strains depending on a single copy of the *tufA499* allele, encoding a mutant form of EF-Tu, Gln125Arg [7], have an extremely slow growth rate even in rich medium [8]. This mutant EF-Tu has a reduced affinity for aa-tRNA but is otherwise proficient in translation *in vitro* [9].

In an effort to understand the basis of the extreme slow growth phenotype we have previously selected chromosomal mutants which almost completely rescue the growth defect and determined that in the majority of cases they had acquired amino acid substitution mutations in *rne*, the gene for RNase E [8]. Analysis of translation and RNA processing in single and double mutants (*tuf*, *rne*) led us to suggest an explanation for the slow growth rate associated with *tufA499*, and its reversal by mutations in *rne* [8]. Thus, mutant EF-Tu, defective in aa-tRNA binding, reduces the saturation of the ribosome by ternary complex, causing the ribosome following the RNA polymerase to pause, probably in a codon-specific manner, exposing the nascent mRNA to RNase E cleavage. Normal growth rate could be restored to the mutant strain either by increasing the total activity of EF-Tu or by reducing the specific activity of RNase E [8]. The *tufA499* mutation apparently initiated a vicious cycle in which a reduced specific activity of the EF-Tu protein was coupled with reduced production of EF-Tu because of increased RNase E-mediated cleavage of *tuf* mRNA.

Here we asked whether the extreme growth defect associated with *tufA499* could be rescued by overexpression of a wild-type gene other than *tuf*. The aim was to gain further insights into the nature of the mutant growth defect and its possible connections with normal growth rate regulation.

## Results

### Identification of plasmid clones that improve the growth rate of TH7509

A P22 phage lysate grown on a *S.* Typhimurium LT2 genomic library made in pBR328 (Experimental Procedures) was used to transduce TH7509 (Table 1) carrying the mutation *tufA499* [8]. Selection plates were screened visually for faster growing transductants. Seven colonies were chosen for further analysis based on their apparent faster growth rate. DNA sequencing of the inserts in plasmids purified from each of the faster-growing clones revealed that five of the seven plasmids carried different overlapping inserts corresponding to nts 3927882–3942536 in the LT2 genome [10]. We decided to focus on these clones. The five clones differed from each other but carried in common the complete sequences of three genes: *spoT, spoU, recG*. This coincidence suggested that one or more of these genes were responsible for the improved growth rate of the *tufA499* mutant strain. These genes are part of the same operon (*gmk – rpoZ – spoT – spoU - recG*), and encode a ppGpp synthetase/hydrolase: *spoT* [11]; a tRNA methyltransferase: *spoU* [12], and an RNA helicase: *recG* [13], respectively.

**Table 1:** Bacterial strains.

Strain	Genotype
TH673	*metA22 metE551 galE496 rpsL120 xyl-404 (Fels2-) Hlnb nml- H2 enx hsdL6 hsdA29 ilv proB1657*::Tn*10 srl-203*::Tn*10*d-Cam *recA1*
TH4527	*Salmonella enterica* subsp. *enterica* serovar Typhimurium strain LT2 wild-type
TH7480	*tufB*::FRT^a^ *trpE91/F′128 pro* ^+^ *lac* ^+^ *zzf-1831*::Tn*10dspc*
TH7483	*tufA499 tufB*::FRT^a^ *trpE91/F′128 pro* ^+^ *lac* ^+^ *zzf-1831*::Tn*10dspc*
TH7507	*tufB*::FRT^a^ *trpE91*
TH7509	*tufA499 tufB*::FRT^a^ *trpE91*
TH7960	*tufB*::FRT^a^ *trpE91*/pBAD TOPO (*spoT*)
TH7963	*tufA499 tufB*::FRT^a^ *trpE91*/pBAD TOPO (empty vector)
TH7964	*tufA499 tufB*::FRT^a^ *trpE91*/pBAD TOPO (*spoT*)
TH7965	*tufA499 tufB*::FRT^a^ *trpE91*/pBAD TOPO (*spoU*)
TH7966	*tufA499 tufB*::FRT^a^ *trpE91*/pBAD TOPO (*recG*)
TH7975	*tufA499 tufB*::FRT^a^ *trpE91 relA21*::Tn*10*
TH7976	*tufB*::FRT^a^ *trpE91 relA21*::Tn*10*
TH7991	*tufA499 tufB*::FRT^a^ *trpE91*/pBAD TOPO (*csgD* 70 nt fragment)
TH8132	*tufB*::FRT^a^ *trpE91 dksA*::KanR
TH8133	*tufA499 tufB*::FRT^a^ *trpE91 dksA*::KanR
TH8385	*tufA499 tufB*::FRT^a^ *trpE91 relA21*::Tn*10*/pBAD TOPO (*spoT*)
TH8386	*tufB*::FRT^a^ *trpE91 relA21*::Tn*10*/pBAD TOPO (*spoT*)
TH8634	*tufA499 tufB*::FRT^a^ *trpE91 relA21*::Tn*10/F′128 pro* ^+^ *lac* ^+^ *zzf-1831*::Tn*10dspc*
TH8635	*tufB*::FRT^a^ *trpE91 relA21*::Tn*10/F′128 pro* ^+^ *lac* ^+^ *zzf-1831*::Tn*10dspc*

a The coding sequence of *tufB* was precisely replaced with an FRT sequence by λ-Red-mediated chromosome recombineering [43], [44].

### Controlled overexpression of *spoT*, *spoU* and *recG*

To determine whether any of the three genes was individually responsible for the observed growth compensation, each of them was amplified by PCR and sub-cloned into a pBAD TOPO vector under the control of an arabinose-inducible promoter (Experimental Procedures). The different plasmid constructs were then introduced into the slow-growing *tufA499* strain TH7509. As negative controls both the empty vector and the vector carrying a non-coding 70 nt sequence (from upstream of the gene *csgD*) were also introduced.

**Figure 1:**
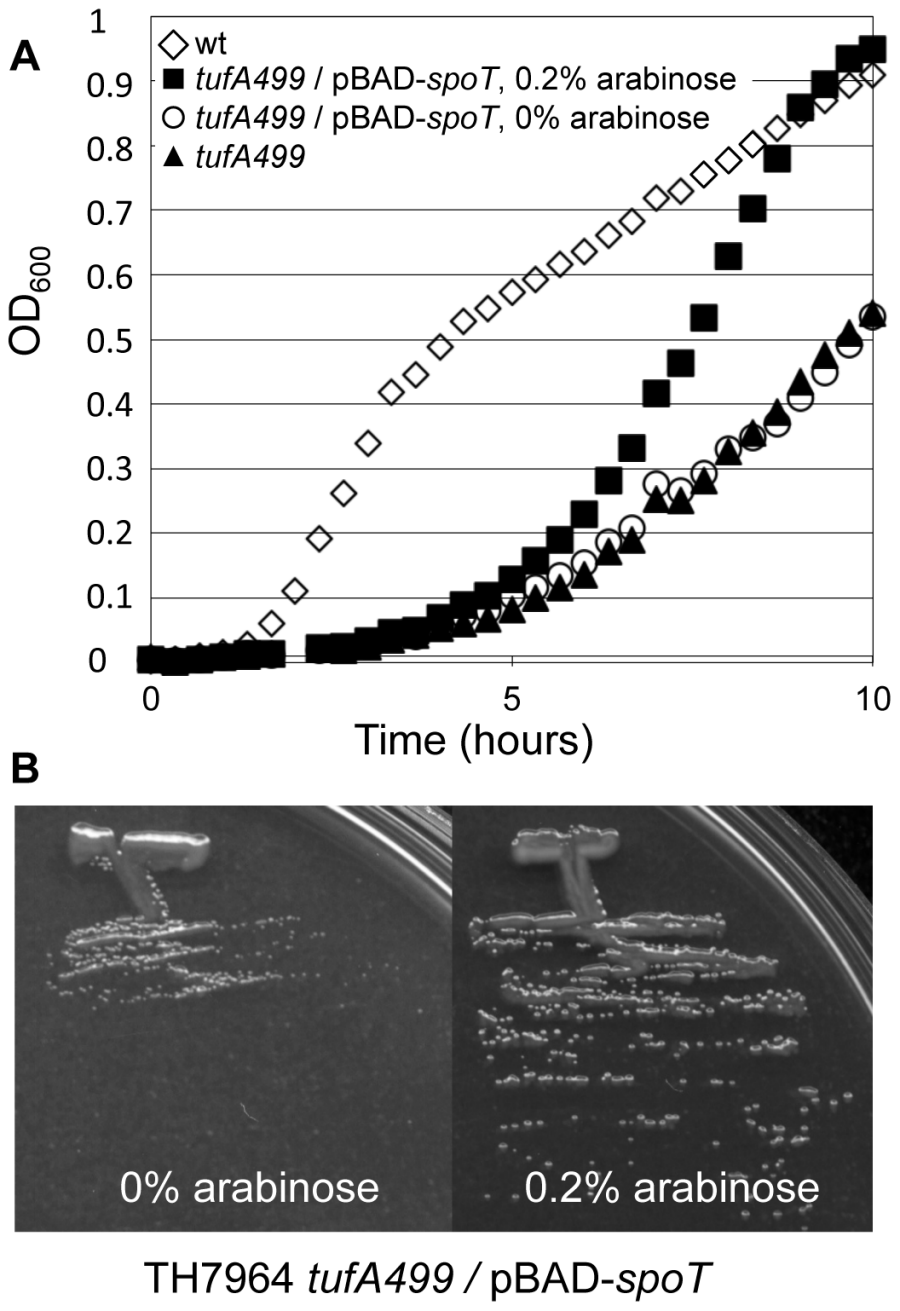
Overexpression of *spoT* increases mutant growth rate and growth yield. (A) Growth as a function of overexpression of *spoT*. TH7507 (*tufA+*), TH7509 (*tufA499*), and TH7964 (*tufA499*/pBAD-*spoT*) grown with or without added arabinose (0.2%) in LB. Growth curves are from a single, representative experiment. (B) TH7964 (*tufA499*/pBAD-*spoT*) grown on LA plates for 16 h at 37°C. Left panel: no arabinose added; Right panel: 0.2% arabinose added to induce expression.

The growth of each of the strains was assayed in liquid medium in the absence and presence of the inducer L-arabinose. Only one strain showed an increase in exponential growth rate and a higher growth yield in the presence of arabinose: TH7964 carrying the mutant allele *tufA499* and the plasmid pBAD-*spoT* (Figure 1A). The improvement in growth yield as a function of the presence of arabinose was also visualized as faster colony growth rate on rich agar medium (Figure 1B). We concluded that overexpression of wild-type *spoT* partially reversed the extreme growth defect associated with *tufA499*. The longer lag-phase associated with the TH7964 strain, even in the presence of arabinose, is not compensated by overexpression of *spoT*.

### Overexpression of *spoT* reduces ppGpp levels in the mutant strain

**Figure 2:**
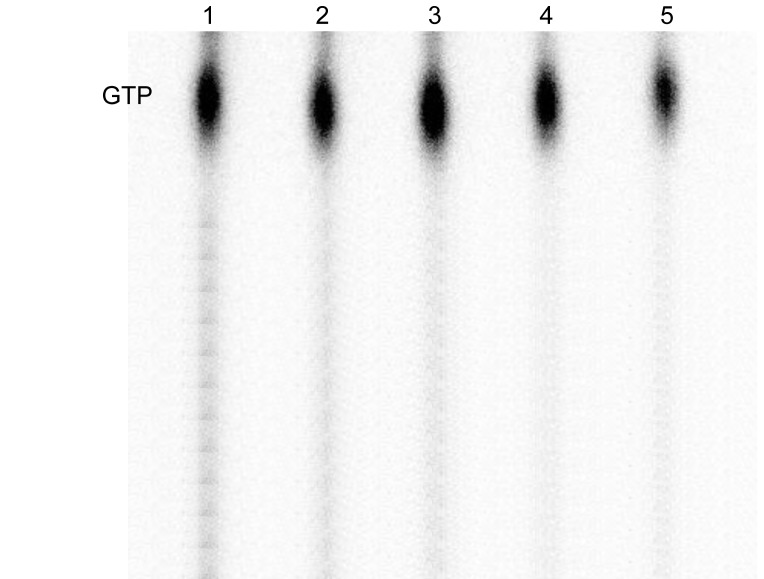
Reduction in ppGpp levels associated with induction of *spoT* overexpression. Thin layer chromatography of guanine nucleotides isolated from TH7964 (*tufA499*/pBAD-*spoT*) grown with different concentrations of arabinose as indicated. The positions of ppGpp, pppGpp and GTP are indicated.

The enzyme encoded by *spoT* is associated with two different activities: ppGpp synthetase and ppGpp hydrolase activity [11], [14]. We asked whether the induction of *spoT* was associated with either an increased or a decreased level of ppGpp in the mutant strain. Cultures of the mutant carrying the plasmid pBAD-*spoT* were grown in rich medium with different levels of arabinose. After overnight growth cultures were harvested and assayed for ppGpp. The level of ppGpp was highest in the uninduced culture and decreased by approximately 80% in fully induced cultures (Figure 2). Thus, overexpression of *spoT* significantly reduces the level of ppGpp in the mutant cell and this reduction is associated with increased growth rate (Figure 1).

### Inactivation of *relA* decreases ppGpp level and increases growth rate

Based on the effect of *spoT* overexpression in reducing ppGpp level, we hypothesized that inactivation of *relA*, which encodes the RelA ppGpp synthetase, would also improve the growth rate of a strain dependent on *tufA499* for protein synthesis.

**Figure 3:**
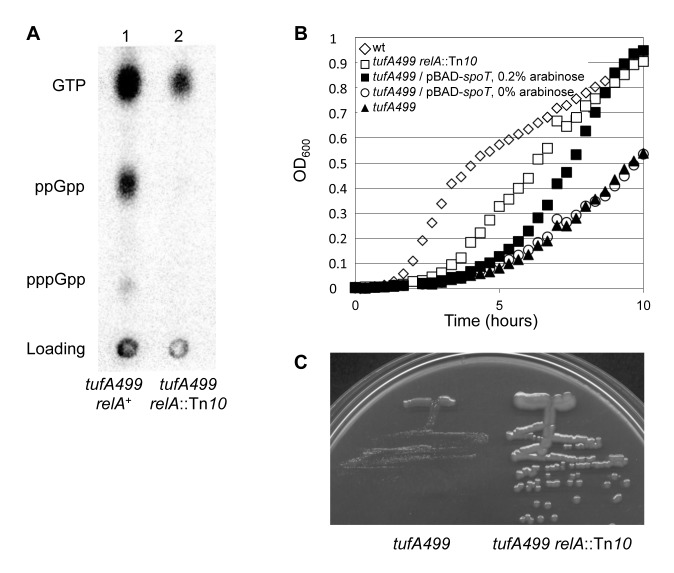
Inactivation of *relA* reduces ppGpp level and increases mutant growth rate. (A) Reduction in ppGpp levels associated with inactivation of *relA*. Thin layer chromatography of guanine nucleotides isolated from strains with the *tufA499* allele. Lane 1: TH7509 (*tufA499*). Lane 2: TH7975 (*tufA499*, *relA21*::Tn*10*). The positions of ppGpp, pppGpp and GTP are indicated. (B) Growth curves of TH7507 (*tufA+*), TH7975 (*tufA499 relA*::Tn*10*), TH7964 (*tufA499*/pBAD-*spoT*) grown with 0.2% arabinose to cause overexpression of *spoT* or 0% arabinose as a control, and TH7509 (*tufA499*), all grown in LB (Bioscreen). Growth curves are from a single, representative, experiment. (C) Strains grown on an LA plate for 18 h at 37°C. Left panel: TH7509 (*tufA499*); Right panel: TH7975 (*tufA499 relA21*::Tn*10*).

We constructed a strain (TH7975) carrying *tufA499* as the only active *tuf* gene and with *relA* inactivated by a transposon insertion (*relA21*::Tn*10*). As expected, the basal level of ppGpp in TH7975 was reduced significantly relative to the level found in TH7509, carrying *tufA499* and wild-type *relA* (Figure 3A). TH7975 also had a significantly higher growth yield and faster growth rate than the isogenic TH7509 (Figure 3B). The positive effect of *relA* inactivation on growth rate was greater than that associated with the overexpression of *spoT* and greatly increased colony growth rate (Figure 3C). We concluded from this experiment that, irrespective of how it was achieved, a reduction in the level of ppGpp in a strain that depends on *tufA499* for production of EF-Tu to drive protein synthesis, resulted in a significant improvement in growth rate. Combining the overexpression of *spoT* with inactivation of *relA* by growing a strain with *relA21*::Tn*10* carrying the pBAD-*spoT* plasmid (TH8385) on 0.2% L-arabinose, did not result in any further increase in colony size, compared to the size of the *tufA499 relA21*::Tn*10* strain TH7975 (data not shown).

### Basal levels of ppGpp differ in mutant and wild-type

Because of the inverse correlation between growth rate of the *tufA499* mutant and level of ppGpp noted above, we decided to compare the basal levels of ppGpp during exponential growth in the wild-type and the mutant. This was done by quantifying the incorporation of radioactive orthophosphate into ppGpp *in vivo*, taking samples throughout the logarithmic growth phase. The incorporation of radioactive orthophosphate into ppGpp was consistently higher, by a factor of 2, in the mutant strain relative to the wild-type: 4.10×10^4^ for the *tufA499* mutant, compared to 1.96×10^4^ for the wild-type (the values are means of three time points taken at different culture ODs during early exponential growth, with three independent experiments for each strain). We concluded that the *tufA499* mutation is associated with unusually high basal levels of ppGpp during exponential growth.

### Inactivation of *dksA* does not improve mutant growth rate

In *E. coli* the protein DksA binds to RNA polymerase and it has been suggested that it acts as a co-factor, sensitizing the polymerase to changes in the cellular levels of ppGpp [15], [16] possibly by stabilizing the ppGpp-RNA polymerase complex [17]. However, the exact relationship between DksA and ppGpp may be more complex because in *E. coli* the absence of ppGpp or DksA exerts opposite phenotypes on cell adhesion [18]. In addition, a transcriptomic analysis of gene expression in *E. coli* deficient in ppGpp or DksA found that many genes were oppositely affected, showing that the regulation of gene expression by ppGpp can in some cases be independent of DksA [19]. To test whether the slow growth phenotype of the EF-Tu mutant would be reversed in the absence of DksA we inactivated the gene by insertion of a kanamycin resistance cassette. Apart from causing a slight reduction in growth rate (also observed in an isogenic strain with a wild-type *tufA* gene) loss of DksA activity did not increase the growth rate of the *tufA499* mutant strain (Table 2). Thus, the effects of altered ppGpp levels on mutant growth rate are not dependent on DksA activity.

**Table 2:** Inactivating *dksA* does not compensate for *tufA499*.

Strain	Genotype	Dt±sd^a^	N
TH7507	*tufA tufB*::FRT	23.4±2.4	27
TH8132	*tufA tufB*::FRT *dksA*::KanR	24.4±1.1	9
TH7509	*tufA499 tufB*::FRT	69.8±11.6	30
TH8133	*tufA499 tufB*::FRT *dksA*::KanR	71.6±6.2	12

a Dt is doubling time of the bacterial cultures, ± standard deviation.

### Inactivation of *relA* does not decrease protein synthesis step-time in the mutant

Although the major regulatory effect of ppGpp is through its interaction with RNA polymerase, ppGpp can also interact with the guanine-nucleotide-binding translation factors IF-2, EF-G and EF-Tu [20] and it was shown, using an *in vitro* translation system, that competition by ppGpp for binding to EF-Tu and EF-G could reduce translation elongation rate [21]. The step time for β-galactosidase synthesis is significantly increased by *tufA499* [8], raising the question of whether the ppGpp effects on mutant growth rate and step-time are mediated through effects on transcription and/or translation. To test this we measured step-times for β-galactosidase synthesis in four isogenic strains (TH7480, TH7483, TH8634 and TH8635) carrying wild-type or mutant *tuf*, and with different basal levels of ppGpp due to the presence of a wild-type or inactivated copy of *relA*. The step-times after induction of *lacZ* were as expected significantly dependent on whether the *tuf* gene was mutant or wild-type, but they did not differ significantly as a function of *relA* activity (Table 3). This result is consistent with the major effects of ppGpp on mutant growth rate and protein synthesis step-time being primarily mediated through transcription rather than translation.

**Table 3:** Inactivating *relA* does not alter step-time of the *tufA499* mutant.

Strain	Genotype^a^	Step-time^b^	n	P^c^	P^d^
TH7480	*tufA tufB*::FRT	106±6.5	4		
TH8635	*tufA tufB*::FRT *relA21*::Tn*10*	111±7	3	0.4035	0.4035
TH7483	*tufA499 tufB*::FRT	198±26	4	0.0009	
TH8634	*tufA499 tufB*::FRT *relA21*::Tn*10*	180±29	6	0.0017	0.3980

a All strains carried the F-factor *F′128 pro* ^+^ *lac* ^+^ *zzf-1831*::Tn*10d-spc*.

b **Step time ± standard deviation (sec).**

c p-values calculated by unpaired t-tests, comparing the step-time of the mutant strains to the TH7480 *tufA* wild-type.

d p-values calculated by unpaired t-tests, comparing *relA21*::Tn*10* strains to the corresponding *relA*+ strain.

### Increased ppGpp level in the *tufA499* mutant is reflected in decreased expression of 16S rRNA

Because ppGpp negatively regulates transcription of ribosomal RNA genes [22] we asked whether the increased basal level of ppGpp in the *tufA499* mutant strain was sufficient to reduce transcription of 16S rRNA. RNA was prepared from exponentially growing cultures of the wild-type strain (TH7507), an isogenic strain with inactivated *relA* (TH7976), the slow-growing *tufA499* mutant (TH7509), and an isogenic strain carrying *tufA499* and inactivated *relA* (TH7975). Relative transcription levels of 16S RNA and tmRNA (used as a standard) were measured by quantitative real-time PCR. The 16S rRNA level in the slow-growing *tufA499* strain was significantly reduced (to 44% of the wild-type level) but was restored back to the wild-type level in the strain carrying both *tufA499* and inactivated *relA* (Table 4). These data are in agreement with the direct measurements of different ppGpp levels in these strains and show that the increased basal level of ppGpp associated with *tufA499* is sufficiently high to negatively regulate transcription of 16S rRNA.

**Table 4:** The *tufA499* mutation is associated with a low expression of 16S rRNA, which can be compensated for by inactivation of *relA*.

Strain	Genotype	Relative 16S expression ±sd^a^	n^b^	p-value^c^
TH7507	*tufB*::FRT	1.01±0.23	6	
TH7976	*tufB*::FRT *relA21*::Tn*10*	0.79±0.05	5	0.0934
TH7509	*tufA499 tufB*::FRT	0.44±0.13	6	0.0007
TH7975	*tufA499 tufB*::FRT *relA21*::Tn*10*	1.25±0.48	5	0.3454

a Relative quantity of 16S rRNA expression compared to tmRNA expression.

b Number of independent RNA preparations.

c p-values calculated by unpaired t-tests, comparing strains to TH7507.

### The *tufA499* mutant shows decreased expression of four tRNA aminoacyl synthetases

**Figure 4:**
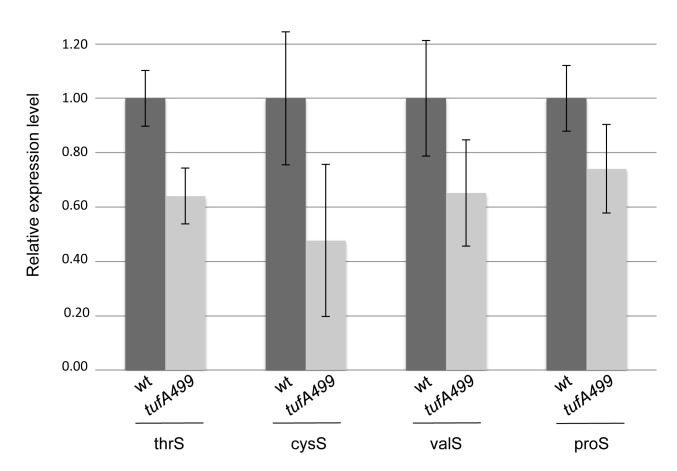
The *tufA499* mutation is associated with a reduced expression of four tRNA aminoacyl synthetases. Expression levels of the synthetase genes *thrS*, *cysS*, *valS* and *proS* were measured by quantitative real-time PCR in wild-type TH7507 (*tufA*+) and TH7509 (*tufA499*). Values are averages of six independent replicates and normalized to the wild-type levels. Standard deviations represented as error bars. The differences between the mRNA levels in the wild-type and the *tufA499* strains are statistically significant according to an unpaired t-test, thrS: p = 0.0005, cysS: p = 0.0105, valS: p = 0.0225, proS: p = 0.0169.

The *tufA499* mutant has previously been shown to cause increased RNase E cleavage of mRNA [8]. Since RNase E is well-known to regulate the expression of several genes, including aminoacyl-tRNA synthetases, via mRNA cleavage [23], we asked if the strain TH7509, carrying *tufA499* as its only *tuf* gene, had an altered expression of tRNA synthetase genes compared to the wild-type. RNA from exponentially growing cultures of the wild-type strain (TH7507) and the slow-growing *tufA499* mutant (TH7509) was prepared. Transcript levels of six different tRNA aminoacyl synthetase genes, *thrS*, *cysS*, *asnC*, *valS*, *proS* and *tyrS*, relative to tmRNA, were measured by quantitative real-time PCR (Materials and Methods). The expression levels of four of these synthetase genes, *thrS*, *cysS*, *valS* and *proS* were significantly reduced in the *tufA499* strain compared to the wild-type strain: down to 50–75% of the wild-type levels (Figure 4).

### Proline tRNAs are aminoacylated to a lower level in the slow-growing *tufA499* mutant

**Figure 5:**
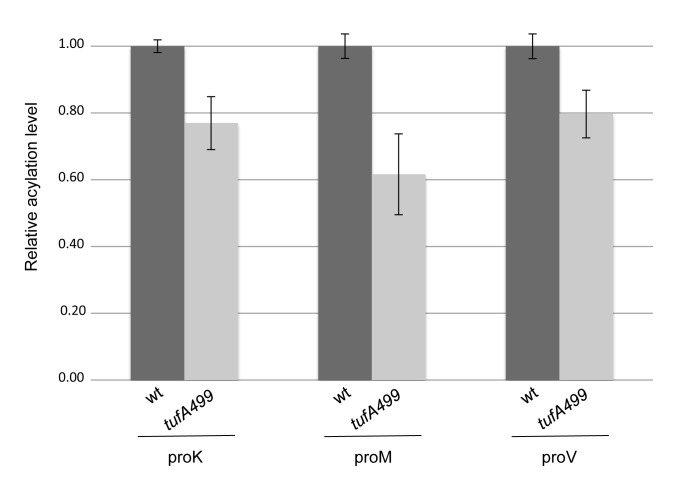
Proline tRNAs are less acylated in the slow-growing *tufA499* mutant. Northern blot measurements of aminoacylation levels of the proline isoacceptors, in wild-type (TH7507) and *tufA499* mutant (TH7509). Values are averages of four or five independent measurements and normalized to the wild-type, standard deviation shown as error bars. The differences between the wild-type and the *tufA499* strains are statistically significant according to an unpaired t-test, proK: p = 0.0007, proM: p = 0.0010, proV: p = 0.0027.

Next, we asked if this reduction in tRNA synthetase mRNA expression was associated with any reduction in the aminoacylation levels of the tRNAs charged by the synthetases with reduced mRNA levels. To examine this, RNA from mid-exponential cultures of wild-type (TH7507) and the *tufA499* mutant (TH7509) was prepared under acidic conditions and analyzed by Northern blotting (Materials and Methods). The relative levels of aminoacylation for the each of the tRNA isoacceptor species were compared in the wild-type strain and the *tufA499* strain. The charging levels of the Thr, Cys, and Val tRNAs showed no difference between mutant and wild-type (data not shown), but all three of the proline tRNAs had a significantly decreased level of aminoacylation in the *tufA499* mutant: 60–80% of the wild-type levels (Figure 5). This confirms that the slow-growing *tufA499* strain is associated with a lower tRNA acylation level for at least one amino acid.

## Discussion

Free-living bacteria constantly adjust their rates and patterns of macromolecular synthesis in response to the nutritional status of their environment [24], [25]. This ability to make appropriate adjustments is key to their survival in natural environments and understanding the details of these processes may also be key to manipulating or controlling bacterial growth and persistence in clinical settings. Under conditions of exponential growth in rich media, *S.* Typhimurium, like its close relative *E. coli*, contains tens of thousands of ribosomes per cell [24]. Under such nutritionally rich conditions the major activity of RNA polymerase is transcription of the 7 rRNA operons and the parts of the genome closely associated with the translation apparatus [24]. If nutritional conditions deteriorate the bacteria can rapidly adjust their transcriptional pattern, directing RNA polymerase away from ribosomal RNA transcription, and favouring transcription of genes and operons required for the biosynthesis of cellular building blocks such as amino acids and nucleotides. A key player in directing and modulating these changes in the pattern of transcription is the guanine nucleotide ppGpp [16], an alarmone and global regulator of transcription [22], [26]. The molecule ppGpp binds to RNA polymerase at the interface of the β′ and the ω subunits, about 30 Å from the active site [27], [28]. The protein DksA also binds directly to RNA polymerase and acts as co-factor to modulate the interaction of ppGpp with RNA polymerase [15], [17], [29] although some genes are independently regulated by ppGpp or DksA [18], [19], [30].

The production and degradation of ppGpp in *S.* Typhimurium and *E. coli* involves two separate genes: *relA* and *spoT* [reviewed in [22], [26]]. RelA has ppGpp synthetase activity only, whereas SpoT can have either ppGpp synthetase or ppGpp hydrolase activities [31] and the level of ppGpp under any particular growth condition depends of the balance of these three activities. The accumulation of ppGpp in the cell leads to a reduction of ribosome synthesis and thus to a reduction in growth rate, via different mechanisms that depend on the type of starvation signal. Starvation for single amino acids activates the RelA ppGpp synthetase [32] whereas starvation for multiple amino acids or for carbon or energy inactivates the SpoT hydrolase, which results in an increase in ppGpp level because of a reduced rate of degradation [31]. Under conditions of exponential growth RelA synthetase is nearly inactive, SpoT hydrolase maintains a constant low activity, and SpoT ppGpp synthetase activity varies in response to the supply of nutrients in the medium to adjust the basal level of ppGpp and thus the rate of ribosome synthesis [31]. This feedback system ensures that the rate of ribosome function (peptide chain elongation rate) is maintained close to the maximum appropriate for the particular nutritional conditions.

In this paper we have shown that bacteria that depend on the mutant allele *tufA499* as the sole source of EF-Tu have an unusually high level of ppGpp synthesis under conditions of logarithmic growth in rich medium and grow very slowly. We also show that genetic alterations that reduce the level of ppGpp in the mutant strain, either by overexpression of *spoT*, or by inactivation of *relA*, increase bacterial growth rate and yield. Overexpression of *spoT* from multicopy plasmids [31], [33], [34] has previously been observed to reduce basal levels of ppGpp and it has been suggested, counter-intuitively, that this reduction is caused by a reduced synthetase activity, due to inactivation of excess SpoT proteins, rather than an increase in hydrolase activity [31]. Regardless of the actual mechanism, our data shows that controlled overexpression of *spoT* causes a decrease in ppGpp levels in response to induction, and increases the growth rate of a strain dependent on *tufA499* for production of EF-Tu. A similar phenotype, with regard to ppGpp levels and growth rate improvement, was also achieved by inactivation of the chromosomal *relA* gene. In a strain with *relA* inactivated, and carrying the pBAD-*spoT* plasmid, growth rate and yield was the same plus or minus arabinose. This shows that the positive effect on growth of inducing *spoT* requires the presence of an active *relA* gene. Inactivation of *dksA* did not increase the growth rate of the *tufA499* mutant strain, suggesting that the influence of ppGpp levels on growth rate of this mutant is independent of DksA activity. We found no evidence that differences in protein synthesis rate were dependent on ppGpp level. However, there was a strong effect of ppGpp level on rRNA transcription. These data support the hypothesis that the effects of ppGpp on protein synthesis and growth rate in the mutant strain are mediated through effects on transcription. In summary, our data show that the growth rate of a strain dependent on *tufA499* can be increased, by reducing the level of ppGpp. The greatest effect is caused by inactivating *relA*. RelA-mediated ppGpp production is dependent on deacylated tRNA entering the ribosomal A-site [35], [36], [37]. Accordingly, the *tufA499* mutation must cause an increase in the amount of deacylated tRNA in the cell sufficient to stimulate a RelA response. Here we measured an increase in the relative level of deacylated proline tRNAs in the *tuf* mutant strain relative to the wild-type (Figure 5, and Figure S1), thus providing evidence of a mechanism to account for the RelA-dependent increased level of ppGpp in the mutant strain.

The actual cause of the increased level of deacylated tRNA associated with *tufA499* is not certain. One possibility is that the weak affinity of the mutant EF-Tu for aminoacyl-tRNAs [9] exposes aminoacylated-tRNA to an increased rate of deacylation before it can enter into the ternary complex [38]. However, another possibility is suggested by our observation that the level of several different tRNA synthetase transcripts, including proS, is lower in the mutant strain (Figure 4). Thus, the reduction in tRNA synthetase transcript level might lead to a reduced rate of tRNA aminoacylation with a consequent increase in the relative level of deacylated tRNAs. Regardless of the exact mechanism, whether due to an increased rate of deacylation or a reduced rate of acylation, the increase in the level of some deacylated tRNA species provides a plausible mechanism for the increased level of ppGpp associated with slow growth in the *tufA499* mutant strain.

## Materials and Methods

### Bacterial strains and growth conditions

All bacterial strains are isogenic with *S.* Typhimurium strain LT2 and are listed in Table 1. Bacteria were grown in Luria broth (LB) and on Luria agar (LA) with incubation at 37°C. Where noted the growth medium was supplemented with ampicillin (100 µg/ml), tetracycline (15 µg/ml), L-arabinose (0.2%). Bacteriophage P22 HT *int* [39] was used to move chromosomal DNA or plasmids by transduction.

### Growth rate measurements

Growth measurements were made in Honeycomb microtiter plates using a Bioscreen C machine (Oy Growth Curves Ab Ltd) to monitor changes in optical density. Single colonies were dissolved in 0.9% NaCl and diluted to OD_600_≈0.15 (∼4×10^7^ CFU/ml). Each well was inoculated with 300 µl media (20 µl of cell mixture added to 280 µl LB, containing L-arabinose and ampicillin as appropriate), equivalent to ∼8×10^5^ CFU/well at time zero. Cultures were grown at 37°C with continuous shaking and OD was monitored every 5 minutes up to 16 hours.

### Step-time measurements

Liquid cultures (20 ml LB, 0.2% glycerol) were set up from bacterial colonies grown on LA plates supplemented with glycerol and spectinomycin (50 µg/ml) and grown to mid-log phase at 37°C. Before induction, a time zero sample (500 µl) was taken and added to 750 µl ice-cold chloramphenicol (0.5 mg/ml in 1∶1 H_2_O∶ethanol). Expression of *lacZ* from the F′128 plasmid was induced by the addition of 200 µl IPTG (0.1 M, final concentration 1 mM). Samples (500 µl) were taken after the induction, either every 10 seconds for 300 seconds (for TH7480 and TH8635) or every 30 seconds for 900 seconds (for TH7483 and TH8634) and added to 750 µl chloramphenicol solution. Cells were pelleted by centrifugation (3 min, 12000 g) and resuspended in 500 ml Z-buffer (0.06 M Na_2_HPO4•2H_2_O, 0.04 M NaH_2_PO_4_•H_2_O, 0.1 M KCl, 0.001 M MgSO_4_•7H_2_O, 0.05 M β-mercaptoethanol). To each sample, 100 µl chloroform and 50 ml 0.1% SDS were added. The tubes were vortexed and left on ice for 20 min to allow the chloroform to sink before 200 µl of each sample were added to a Honeycomb plate with 40 µl ONPG (4 mg/ml) added per well. The plate was run in a Bioscreen C machine (Oy Growth Curves Ab Ltd) and absorbance at 420 nm and 540 nm was measured. Background absorbance (ONPG in Z-buffer without cells) and absorbance at time zero were subtracted and the data were plotted with √((OD_420_)−(1.75*OD_540_)) as a function of time. The intercept with the x-axis of the induced curve is the step-time, the time it takes to produce the first β-galactosidase activity.

### Plasmids

An *S.* Typhimurium LT2 DNA library, generated by partial digestion of chromosomal DNA with Sau3A cloned into the BamHI site of pBR328 was obtained from Dan Andersson, Uppsala University. A P22 lysate made on the library was transduced into the slow-growing *tufA499* mutant TH7509, with selection for plasmid-encoded ampicillin resistance on LA ampicillin agar. The pBR328 (empty) vector was used as a negative control. Transductants were screened visually for faster-growing colonies. Lysates made on candidate fast-growing transductants were used to confirm the linkage between the plasmid and the growth compensation phenotype by back-crossing into TH7509. Plasmids were purified from 7 different transductants that conferred an apparent growth advantage on TH7509, using the QIAprep Spin Miniprep Kit (Qiagen), and the inserts were identified by DNA sequencing (Macrogen Inc, Korea).

The individual genes of interest (*spoT*, *spoU*, and *recG*) were PCR amplified from *S.* Typhimurium LT2 chromosomal DNA using primers (Table 5) such that the amplified sequence began with the start codon and ended approximately 6 nts after the termination codon of each gene. A non-coding 70-nucleotide fragment corresponding to the 5′UTR of the gene *csgD*, was PCR amplified from the plasmid pEH87 (E. Holmqvist, Uppsala University) for use as a negative control. All PCR products were cloned into the pBAD TOPO vector (Invitrogen) according to the manufacturers' instructions for TOPO TA cloning. Constructs were confirmed by DNA sequencing (Macrogen Inc, Korea). pBAD TOPO plasmids with the correct insertions were purified using the QIAprep Spin Miniprep Kit (Qiagen) and transformed into the restriction-minus *S.* Typhimurium strain TH673 by electroporation. Plasmids prepared from TH673 strain were then electroporated into the strain TH7509 (*tufA499*) for further analysis.

**Table 5:** Oligodeoxyribonucleotide primers.

Primer	Oligonucleotide sequence 5′ - 3′	Primer use
pBR328 fw	CTTCGCTACTTGGAGCCACT	Plasmid insert sequencing
pBR328 rv	GATCTTCCCCATCGGTGAT	Plasmid insert sequencing
spoT Trc fw	TTGTATCTGTTTGAAAGCCTG	PCR of *spoT*
spoT Trc rv	ATAGCGCTAGTTTCGTTACG	PCR of *spoT*
spoU Trc fw	ATGAATCCAAAACGTTATGC	PCR of *spoU*
spoU Trc rv	TGGTGATTACCCTGCCGCCT	PCR of *spoU*
recG Trc fw	ATGTCAGGCCGCTTGTTAGA	PCR of *recG*
recG Trc rv	CGAATAGGATTAGGCGTTGG	PCR of *recG*
EHO-333 fw	GTTGCACTGCTGTGTGTAGT	PCR of *csgD*
EHO-231 rv	CAGATGTAATCCATTAGTTTTATATTTTAC	PCR of *csgD*
ssrA fw	GGCGGTTGGCCTCGTAA	qRT-PCR tmRNA
ssrA rv	GTTATTAAGCTGCTAAAGCGT	qRT-PCR tmRNA
rrsA fw	CCTTACGACCAGGGCTACACA	qRT-PCR 16S RNA
rrsA rv	CTCGCGAGGTCGCTTCTC	qRT-PCR 16S RNA
thrS fw	GACCACCCTGTAAGCCCGAT	qRT-PCR thrS
thrS rv	AAGCATCAACCAGCTCGCCA	qRT-PCR thrS
cysS fw	GTCGCGCTGGTCGACAGAAT	qRT-PCR cysS
cysS rv	GAATATGGTGCGTCGCACGC	qRT-PCR cysS
valS fw	GATAATCTTTACCGTCTGCG	qRT-PCR valS
valS rv	TCTGACCTGGAAGTGGAAAA	qRT-PCR valS
proS fw	TTTTGGCATTTGGCGTATCG	qRT-PCR proS
proS rv	TACGCGGCTAACATTGAACT	qRT-PCR proS
proK Northern	CCTCCGACCCCTTCGTCCCG	Northern blotting proK
proM Northern	CCTCCGACCCACTGGTCCCA	Northern blotting proM
proV Northern	CCTCCGACCCCCGACACCCC	Northern blotting proV

### Polymerase Chain Reaction (PCR)

A bacterial colony was dissolved in 100 µl ddH_2_O and boiled for 5 min. DNA was amplified using PuReTaq Ready-To-Go PCR beads (GE Healthcare), with a final reaction volume of 25 µl including 1 µl DNA solution and 0.4 µM each of forward and reverse primers (Table 5). The PCR reactions were carried out in a PTC-200 Thermocycler (SDS-Diagnostics). To amplify *spoT*, the PCR programme was initiated by denaturation at 95°C for 5 min, followed by 30 cycles of denaturation at 95°C for 30 sec, primer annealing at 54°C for 30 sec and elongation at 72°C for 2 min. The programme was ended with a final 10 min elongation step at 72°C. For *spoU*, the elongation time was 45 sec and for *recG*, the annealing temperature was 58°C. For PCR of the 5′UTR of the gene *csgD*, the annealing temperature was 60°C and the elongation time 30 sec. The PCR products were purified using the QIAquick PCR purification kit (Qiagen).

### Measurement of *in vivo* ppGpp levels by thin layer chromatography (TLC)

The strains TH7507 and TH7509 were initially grown on LA at 37°C for 14–16 hrs to generate actively growing bacterial colonies which were suspended in LB. This procedure was followed to minimize the risk of selecting faster-growing suppressors during growth of the initial culture. The OD_600_ of the suspension was adjusted to 0.01 in pre-warmed LB. Aliquots of 0.5 ml were transferred to cultures tubes (three for each of the time points to be taken) and incubated at 37°C without shaking (for practical reasons of shielding the radioactivity). ^32^P (Perkin Elmer) was added to an activity of 100 µCi/ml culture 30 min before each time sample was taken. Samples were taken with a frequency corresponding to approximately once per cell doubling. Each sample was centrifuged for 1 min at ∼10,000 g to pellet the cells. Cell pellets were resuspended in 1 ml 0.9% NaCl and washed by centrifugation three times. The final pellet was resuspended in 200 µl 0.9% NaCl to which was added 200 µl 20% formic acid to lyse the cells. This mixture was vortexed and stored at −20°C overnight. Before application to TLC plates, cell debris was pelleted by centrifugation at 10,000 g for 5 min at 4°C. 5–100 µl of supernatant (based on the OD_600_ of the cultures) was applied drop-wise onto a TLC PEI Cellulose F membrane (Merck). As a size marker, 0.2 µmol of non-radioactive ppGpp (a gift from Vasili Hauryliuk, Uppsala) was applied onto the same membrane. Chromatography was performed in 1.5 M KH_2_PO_4_ (pH 3.0) until the buffer level had reached the top of the membrane. The marker was visualized under UV-light. The membrane was dried and the chromatography results were visualized using a PhosphorImager (Molecular Dynamics) and quantified with the ImageQuant software, version 4.2a (Molecular Dynamics).

To measure ppGpp as a function of *spoT* overexpression or *relA* inactivation the strains TH7509 (*tufA499*), TH7964 (*tufA499*/pBAD-*spoT*), and TH7975 (*tufA499 relA21*::Tn*10*) were initially grown as colonies on LA as described above (with ampicillin for TH7964), and resuspended in LB to an OD_600_ 0.1. For strain TH7964 the LB was supplemented with ampicillin and a series of suspensions were prepared containing different concentrations of L-arabinose (0, 0.025%, 0.05%, 0.1%, 0.2%). Each culture (0.5 ml) was incubated at 37°C with shaking. After 90 min incubation ^32^P was added to an activity of 100 µCi/ml of culture and incubation was continued for a further 120 min (the OD_600_ was approximately 0.6). Cultures were harvested, prepared and applied to TLC plates as described above.

### RNA preparation and relative quantification of RNA by real-time PCR (rtPCR)

Fresh colonies of TH7507, TH7976, TH7509 and TH7975 grown on LA were inoculated into liquid LB medium and grown for a further 3 cell generations. 0.5 ml samples were extracted and mixed with 1 ml RNA protect Bacteria Reagent (Qiagen). Total RNA was isolated using the RNeasy Mini Kit (Qiagen), all steps according to the manufacturer's instructions. The quality of the RNA was assayed visually by gel electrophoresis, and the concentration of the different samples was measured using a Nanodrop NO-1000 spectrophotometer (Thermo Scientific). To remove chromosomal DNA from the RNA preparations the DNase Turbo DNA-free (Ambion) kit was used according to the manufacturer's instructions. 500 ng RNA was converted into cDNA using the High Capacity Reverse Transcription kit (Applied Biosystems), with RT buffer, dNTP mix, random primers, and reverse transcriptase according to the manufacturer's instruction, in a total reaction volume of 50 µl. The thermal steps used were 10 min at 25°C and 2 hours at 37°C. For quantitative real-time PCR reactions, 5 µl cDNA (diluted 1∶5), 10 µl PerfeCTa SYBR Green FastMix (Quanta Biosciences), 1.25 µl of 6 µM forward and reverse primers, Table 5, (to a final concentration of 0.375 µM), and ddH_2_O was added to a final reaction volume of 20 µl. The Eco Real-Time PCR System (Illumina) was used for running the PCR program and for analyzing the data. The gene *ssrA* (STM2693), encoding tmRNA, was used as a reference in the calculations for relative expression.

### RNA preparation under acidic conditions and Northern blot measurements of aminoacylation levels

Fresh colonies of TH7507 and TH7509 grown on LA were inoculated into liquid LB medium and grown to an OD_600_ of 0.2 (mid-exponential phase). 50 ml of culture were poured into the same volume of 10% trichloroacetic acid and the tubes were transferred to ice [40]. The RNA was prepared essentially as described by [41] and [42]. The culture-TCA mixes were centrifuged, the pellets resuspended in the last drop of supernatant and transferred to six microfuge tubes per 50 ml culture. The cells were pelleted and dissolved in 500 µl NaAc buffer (0.3 M NaAc pH 4.5, 10 mM Na_2_EDTA). RNA was extracted by adding 600 µl phenol (pH 4.3, Sigma Aldrich) to the samples and vortexed repeatedly for 15 min. After centrifugation at 12000 g for 15 min, the aqueous phases were carefully removed to new tubes. The phenol was re-extracted with 250 µl NaAc buffer. RNA was precipitated by the addition of 450 µl (1 volume) ice-cold 99.5% ethanol. Samples were kept at −20°C overnight and centrifuged at 12000 g for 30 min. The RNA pellets were washed twice with 300 µl 70% ethanol and dissolved in NaAc, pH 5.0. In total, the RNA from 50 ml culture was dissolved in 90 µl NaAc.

2.5 µg total RNA was mixed 1∶1 with acid urea sample buffer (0.1 M sodium acetate pH 5.0, 8 M urea, 0.05% bromphenol blue, 0.05% xylene cyanol FF) and loaded on a polyacrylamide gel (8% polyacrylamide [19∶1 acrylamide/bisacrylamide], 0.1 M sodium acetate pH 5.0, 8 M urea, 0.15% TEMED, 0.7% ammonium persulfate). The gel was run at 400 V at 4°C for approximately 18 h. 0.1 M sodium acetate buffer (pH 5.0) was used as running buffer. Transfer of the RNA to a nylon membrane (Hybond-N+, GE Healtcare) was done for 3 h using the Novex semi-dry blotter (Invitrogen). The transfer was conducted at 3 V, 250 mA, with 40 mM Tris-HCl pH 8.0, 2 mM Na_2_EDTA as transfer buffer. RNA was UV-crosslinked to the membrane. The membranes were pre-hybridized (6×SSC, 10× Denhardt's solution, 0.5% SDS) for 5 h at 42°C, rolling in a Hyb-Aid oven. Oligonucleotides were labelled with γ-^32^P-ATP (3000 Ci/mmol, Perkin Elmer), using T4 polynucleotide kinase (Thermo Fisher). Excess ^32^P-ATP was removed by using G50 columns from GE Healthcare. Hybridization of the RNA to ^32^P-labeled DNA oligonucleotides, specific to the tRNA targets (Table 5), was carried out in hybridization buffer (6×SSC, 0.1% SDS, 10^6^ cpm/ml ^32^P-labeled probe) for 12 h, with rolling at 42°C. Stringency washing of the membranes to remove unbound probe was carried out by 2×10 min washes at room temperature in each of 6×SSC, 4×SSC, and 2×SSC. The membranes were visualized using a Personal Molecular Imager (Bio-Rad) and analyzed using Quantity One software (Bio-Rad).

## Supporting Information

Figure S1 **Northern blot measurement of proK tRNA aminoacylation.** RNA was prepared from mid-log phase cultures of wild-type (TH7507) and *tufA499* mutant (TH7509) under acidic conditions, run on a polyacrylamide gel and transferred to nylon membrane. The figure shows a scan of a representative blot of a membrane hybridized with a ^32^P-ATP-labeled probe for the *proK* tRNA, showing a lower level of acylated pro-tRNA in the *tufA499* mutant relative to the wild-type.
(TIF)
