# Supplementary figures and images for: Reducing ppGpp Level Rescues an Extreme Growth Defect Caused by Mutant EF-Tu
Source: PLoS One. 2014 Feb 28;9(2):e90486. doi: 10.1371/journal.pone.0090486 (PMC3938759; doi:10.1371/journal.pone.0090486)

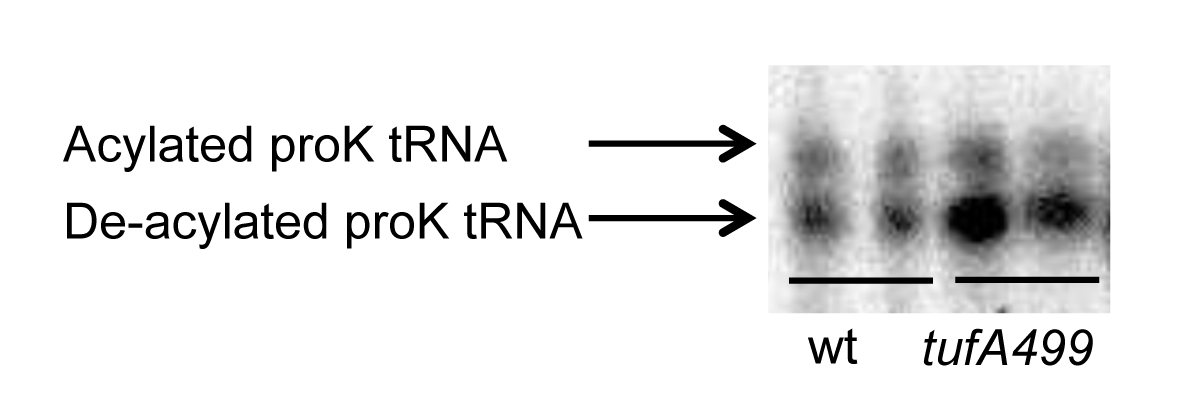

Supplement: Figure S1 — Northern blot measurement of proK tRNA aminoacylation. RNA was prepared from mid-log phase cultures of wild-type (TH7507) and tufA499 mutant (TH7509) under acidic conditions, run on a polyacrylamide gel and transferred to nylon membrane. The figure shows a scan of a representative blot of a membrane hybridized with a 32P-ATP-labeled probe for the proK tRNA, showing a lower level of acylated pro-tRNA in the tufA499 mutant relative to the wild-type. (TIF) [file pone.0090486.s001.tif]
